# Unlocking High-Efficiency Methane Oxidation with Bimetallic Pd–Ce Catalysts under Zeolite Confinement

**DOI:** 10.1021/acsenvironau.3c00008

**Published:** 2023-05-16

**Authors:** Xiaomai Chen, Xuefeng Shi, Peirong Chen, Bowen Liu, Meiyin Liu, Longwen Chen, Daiqi Ye, Xin Tu, Wei Fan, Junliang Wu

**Affiliations:** † National Engineering Laboratory for VOCs Pollution Control Technology and Equipment, Guangdong Provincial Key Laboratory of Atmospheric Environment and Pollution Control, School of Environment and Energy, South China University of Technology, Guangzhou 510006, China; ‡ College of Light Chemical Industry and Materials Engineering, Shunde Polytechnic, Foshan 528333, China; § Department of Electrical Engineering and Electronics, University of Liverpool, Liverpool L69 3GJ, U.K.; ∥ Department of Chemical Engineering, University of Massachusetts—Amherst, Amherst, Massachusetts 01003, United States

**Keywords:** catalytic methane oxidation, silicalite-1 zeolite, bimetallic Pd catalysts, confinement, Pd−Ce interaction

## Abstract

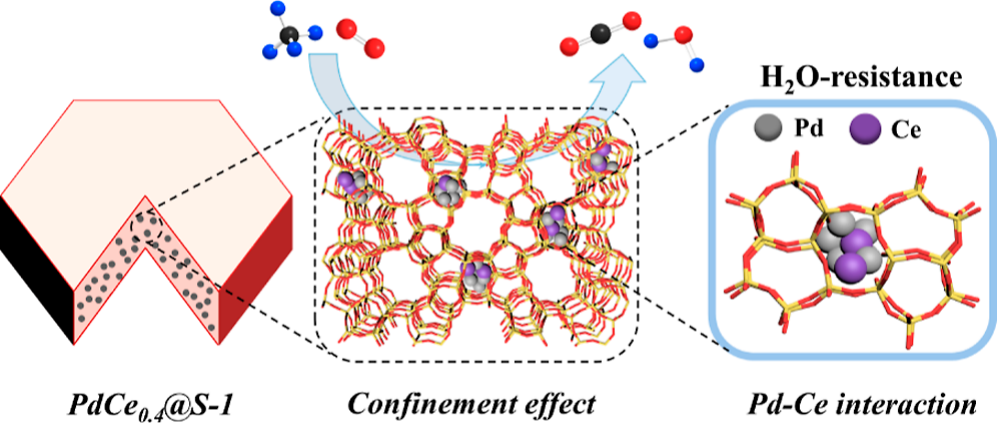

Catalytic complete oxidation is an efficient approach to reducing methane emissions, a significant contributor to global warming. This approach requires active catalysts that are highly resistant to sintering and water vapor. In this work, we demonstrate that Pd nanoparticles confined within silicalite-1 zeolites (Pd@S-1), fabricated using a facile in situ encapsulation strategy, are highly active and stable in catalyzing methane oxidation and are superior to those supported on the S-1 surface due to a confinement effect. The activity of the confined Pd catalysts was further improved by co-confining a suitable amount of Ce within the S-1 zeolite (PdCe_0.4_@S-1), which is attributed to confinement-reinforced Pd–Ce interactions that promote the formation of oxygen vacancies and highly reactive oxygen species. Furthermore, the introduction of Ce improves the hydrophobicity of the S-1 zeolite and, by forming Pd–Ce mixed oxides, inhibits the transformation of the active PdO phase to inactive Pd(OH)_2_ species. Overall, the bimetallic PdCe_0.4_@S-1 catalyst delivers exceptional outstanding activity and durability in complete methane oxidation, even in the presence of water vapor. This study may provide new prospects for the rational design of high-performance and durable Pd catalysts for complete methane oxidation.

## 1 Introduction

Methane (CH_4_) is the main component of natural gas and is extensively used in power generation and other heating applications due to its high energy density and low emission of gaseous pollutants.^1−3^ However, as one of the most predominant greenhouse gases, methane has a global warming potential 105 and 28 times stronger than that of CO_2_ on a 20 year and 100 year scale, respectively.^4,5^ Therefore, increasing attention has been given to strategies for the reduction of unburned methane emissions. Catalytic complete oxidation has been proven to be a promising technology with both environmental and economic benefits in minimizing CH_4_ emissions and the resulting atmospheric pollution.^6−8^ Nonetheless, it remains a great challenge to lower the ignition temperature of CH_4_ oxidation to 400 °C and below, primarily due to the extremely stable and highly symmetrical structure of the CH_4_ molecule.^1,9^

Pd-based catalysts have demonstrated excellent ability to activate C–H bonds of CH_4_ and are among the most active catalysts for complete CH_4_ oxidation.^10−12^ However, the rapid deactivation induced by Pd sintering under real conditions (especially in the presence of steam) is one of the obstacles to the extensive applications of Pd catalysts.^13,14^ To address this, confining ultrasmall Pd nanoparticles (NPs) into microporous zeolites with well-defined channels has been proven to be an effective strategy to suppress the agglomeration and sintering of Pd NPs and to maintain their catalytic performance in CH_4_ oxidation.^15−19^ For example, a Pd catalyst encapsulated in silicate-1 (Pd@S-1) at a low Pd loading of 0.6 wt % achieved a complete combustion temperature (*T*_100_) of 380 °C, as well as excellent water resistance was achieved over a Pd catalyst encapsulated in silicate-1 (Pd@S-1) at a low Pd loading of only 0.6 wt %, due to a strong confinement effect and the high hydrophobicity of the S-1 zeolites.^16^ The introduction of a second nonprecious metal can further promote the activity and stability of Pd catalysts.^20−23^ Shi et al. and co-workers incorporated a series of metals (La, Ce, Sm, Nd, and Tb) into Pd/H-ZSM-5 for methane oxidation and decreased the *T*_100_ by 40 °C in methane oxidation.^24^ They found that Ce incorporation could weaken the Pd–O bond in Pd/H-ZSM-5, resulting in an improved reactivity of surface oxygen species. In addition, the excellent redox properties and oxygen storage capacity of CeO_2_ are also favorable for CH_4_ oxidation.^3,25,26^ Recently, Peng et al. demonstrated that core–shell PdCe@SiO_2_ catalysts displayed both superior activity and outstanding H_2_O and SO_2_ tolerance in CH_4_ oxidation due to the strong interactions between Pd and CeO_2_.^27^

Herein, we developed zeolite-confined bimetallic Pd–Ce catalysts by encapsulating Pd–Ce mixed oxides within hydrophobic S-1 zeolites using a one-pot hydrothermal method to take advantage of the confinement effect and Pd–CeO_2_ interaction in CH_4_ combustion. The resulting PdCe_*x*_@S-1 catalysts exhibited superior performance in CH_4_ oxidation, with increased activity and H_2_O tolerance. Comparative investigations using high-resolution transmission electron microscopy (HRTEM) together with ultramicrotomy, X-ray photoelectron spectroscopy (XPS), O_2_-temperature-programed desorption (O_2_-TPD), and diffuse reflection infrared Fourier transform spectroscopy with CO as the probe molecule (CO-DRIFTS) revealed that the cooperative effects of confinement and Pd–CeO_2_ interactions are crucial for the high performance of the PdCe_*x*_@S-1 catalysts. In situ DRIFTS was used to monitor of the intermediates during CH_4_ oxidation to illustrate the reaction mechanisms over the bimetallic catalysts.

## 2 Experimental Section

### 2.1 Catalyst Preparation

**Figure 1:**
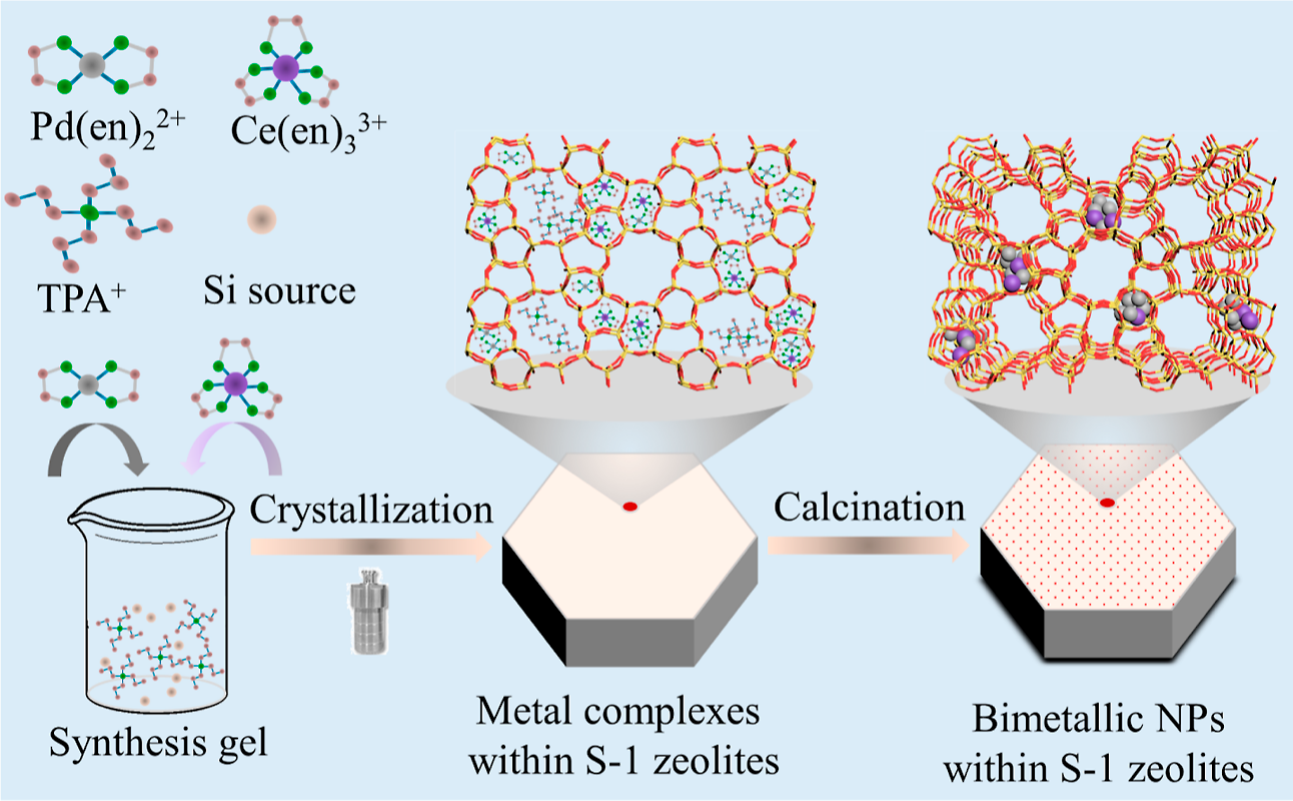
Schematic illustration of the preparation of PdCe_*x*_@S-1.

S-1 zeolite-encapsulated bimetallic Pd–Ce oxides with different Ce/Pd molar ratios were synthesized via a hydrothermal method, as illustrated in Figure 1. For comparison, monometallic Pd@S-1 and Ce@S-1 catalysts were prepared by the same method, and Pd/S-1 and PdCe_0.4_/S-1 samples were synthesized using an incipient wetness impregnation (IWI) method.

The S-1 zeolite was synthesized according to previously published literature.^28^ First, 13 g of tetrapropylammonium hydroxide (TPAOH) solution was mixed with 15.45 g of deionized water at room temperature and stirred for 15 min. Then, 8.32 g of tetraethyl orthosilicate was added dropwise into the above mixture with a continuous stirring for 6 h. Finally, the obtained zeolite precursor gel with a molar ratio of 1 SiO_2_/0.4 TPAOH/35 H_2_O was poured into a 100 mL Teflon-lined stainless-steel autoclave and hydrothermally treated in an oven at 170 °C for 4 days under static conditions. The as-synthesized solid sample was washed by centrifugation with water and ethanol several times and then dried overnight at 80 °C, followed by calcination in air at 550 °C for 8 h at a heating ramp of 1 °C min^–1^.

Before preparing the PdCe_*x*_@S-1 catalyst, a solution of [Pd(NH_2_CH_2_CH_2_NH_2_)_2_]Cl_2_ was prepared by dissolving 0.20 g of PdCl_2_ in a mixture of 1 mL of ethylenediamine and 4 mL of water. Similarly, 0.42 g of CeCl_3_·7H_2_O was added to the same mixture to obtain the [Ce(NH_2_CH_2_CH_2_NH_2_)_3_]Cl_3_ solution. PdCe_*x*_@S-1 and Ce@S-1 were synthesized using the same process as S-1 zeolite, but with the addition of [Pd(NH_2_CH_2_CH_2_NH_2_)_2_]Cl_2_ and/or [Ce(NH_2_CH_2_CH_2_NH_2_)_3_]Cl_3_ to the precursor gel. To prepare PdCe_*x*_@S-1, 1.2 mL of [Pd(NH_2_CH_2_CH_2_NH_2_)_2_]Cl_2_ solution and *x* mL (*x* = 0, 1, 2, 4) of [Ce(NH_2_CH_2_CH_2_NH_2_)_3_]Cl_3_ were added dropwise to the synthesis mixture and stirred continuously for 1 h. Ce@S-1 was synthesized using 1 mL of [Ce(NH_2_CH_2_CH_2_NH_2_)_3_]Cl_3_ under the same conditions. The resulting gel, with a molar ratio of 1.0 SiO_2_/0.4 TPAOH/35 H_2_O: 0.0075 [Pd(NH_2_CH_2_CH_2_NH_2_)_2_]Cl_2_/*y* [Ce(NH_2_CH_2_CH_2_NH_2_)_3_]Cl_3_ (*y* = 0, 0.003, 0.00675, 0.0165) was transferred into a 100 mL Teflon-lined stainless-steel autoclave and heated to 170 °C for 4 days under static conditions. Finally, the as-synthesized catalysts were centrifuged, washed, and dried under the same conditions as the S-1 preparation. The collected solid catalysts were then calcined at 550 °C for 8 h in air.

The Pd/S-1 catalyst was prepared using the IWI method, which involved using a solution of (NH_4_)_2_PdCl_4_. Specially, 1 g of calcined S-1 zeolite was impregnated with 2.67 mL of (NH_4_)_2_PdCl_4_ (0.05 M) and placed in ultrasonication for 2 h to improve the dispersion of the metal on the S-1 zeolite. The resulting suspension was then dried at 80 °C overnight and subsequently calcined at 550 °C in air for 8 h. CeCl_3_·7H_2_O was used as the source of Ce to synthesize PdCe_0.4_/S-1, following the same procedure as for PdCe_*x*_@S-1.

### 2.2 Catalyst Characterization

Comprehensive catalyst characterizations were carried out for all catalysts, including thermal gravimetric analysis (TGA, Figure S1), inductively coupled plasma–mass spectrometry (ICP–MS), powder X-ray diffraction (PXRD), N_2_ physisorption isotherms, thermal gravimetric analysis, TEM, high-angle annular dark-field (HAADF) and elemental mapping, CO chemisorption, XPS, Raman spectroscopy, O_2_-TPD, electron paramagnetic resonance (EPR), methane temperature-programed reaction (CH_4_-TPR), and in situ DRIFTS. Detailed procedures for these characterizations are provided in the Supporting Information.

### 2.3 Catalytic Evaluation

The activities of PdCe_*x*_@S-1 and reference catalysts in CH_4_ combustion were tested at atmospheric pressure using a quartz fixed-bed flow reactor. Typically, 100 mg of catalyst (40–60 mesh) was mixed with 400 mg of quartz sand and placed in the middle of a quartz tube that was blocked by silica wool. The reactant gas containing 1.0 vol % CH_4_, 20 vol % O_2_, and balanced with N_2_ was flowed through the reactor at a total flow rate of 100 mL min^–1^, corresponding to a gas hourly space velocity (GHSV) of 60,000 mL g^–1^ h^–1^. The temperature of the reactor was then programed from room temperature to 440 °C at intervals of 20 °C, with each temperature held for 30 min. The inlet and outlet gas concentrations of CH_4_ were detected by an online gas chromatograph (GC-2014, Japan) equipped with a thermal conductivity detector (TCD) and a flame ionization detector (FID).

The conversion of CH_4_ () was calculated according to the following equations
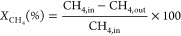
1where CH_4,in_ and CH_4,out_ are the inlet and outlet concentrations of CH_4_, respectively.

Kinetic measurements were further performed by controlling the temperature between 200 and 260 °C to maintain CH_4_ conversions below 15% in order to eliminate the limitations of heat and mass transfer. The CH_4_ reaction rate () and the turnover frequencies (TOF) were estimated using the following equations
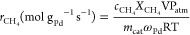
2
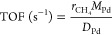
3where  is the CH_4_ concentration in the feed gas, *V* is the total flow rate, *P*_atm_ is the atmospheric pressure (101.3 kPa), *m*_cat_ is the catalyst mass used for the measurement, ω_Pd_ is the Pd loading measured by ICP, *R* is the molar gas constant (8.314 Pa m^3^ mol^–1^ K^–1^), *T* is the temperature (298 K), and *M*_Pd_ is the atomic weight of Pd (106.4 g mol^–1^). *D*_Pd_ is the dispersion of Pd measured by CO chemisorption (Table 1). Moreover, the apparent activation energy (Ea) values were calculated using the Arrhenius equation by plotting ln () vs 1000/*T*.

To evaluate the temperature tolerance of the representative catalysts (PdCe_0.4_@S-1, Pd@S-1, and Pd/S-1), a temperature cycle of 300 → 600 → 300 °C was performed with an interval of 100 °C for 10 h. The stability test of the catalysts was then tested for at least 40 h at 310 °C under dry conditions. In addition, 5.0 vol % water vapor was introduced using a bubbling device to explore the water resistance ability of the catalysts. The GHSV was kept consistent in all tests.

## 3 Results and Discussion

### 3.1 Structural Characterization

The actual Pd and Ce loadings in PdCe_*x*_@S-1 (*x* denotes the Ce/Pd molar ratio), Pd/S-1, and PdCe_0.4_/S-1 were determined by ICP–MS, and the results are summarized in Tables 1 and S1. The Pd contents in all the samples are similar (1.0 wt %), and the Ce/Pd molar ratios are 0.4, 0.9, and 2.2 for PdCe_0.4_@S-1, PdCe_0.9_@S-1, and PdCe_2.2_@S-1, respectively. According to PXRD (Figure S2), all the samples present the typical MFI topology structure (JCPDS 44-0003) and good crystallinity, indicating that the introduction of Pd or Ce phase did not affect the zeolite crystallization. Moreover, no characteristic diffraction peaks of Pd or Ce species were detected, implying good dispersions of Pd and Ce species within the zeolite. The Brunauer–Emmett–Teller (BET) surface areas (Figure S3 and Tables 1 and S2) were found to be similar (ca. 380 ± 10 m^2^ g^–1^) for all the Pd-contained and Pd-free S-1 samples. However, a decrease in micropore volume was noticed in Pd@S-1 (0.07 cm^3^ g^–1^) and PdCe_0.4_@S-1 (0.09 cm^3^ g^–1^) compared with pure S-1 zeolite (0.13 cm^3^ g^–1^), likely due to the partial occupation of the micropores by PdO NPs.^17,28^

**Table 1:** Elemental Composition, Specific Surface Area, Size, and Dispersion of Metal NPs

samples	Pd loading (wt %)^a^	Ce loading (wt %)	*S*_BET_ (m^2^ g^–1^)^b^	Pd dispersion (%)^c^
Pd/S-1	1.07		387	15.2
Pd@S-1	1.00		389	19.7
PdCe_0.4_@S-1	1.09	0.58	386	26.2

a Measured by ICP–MS.

b Obtained from BET.

c Calculated by CO chemisorption (sample was pretreated at 300 °C for 1 h in 10 vol % H_2_/Ar).

**Figure 2:**
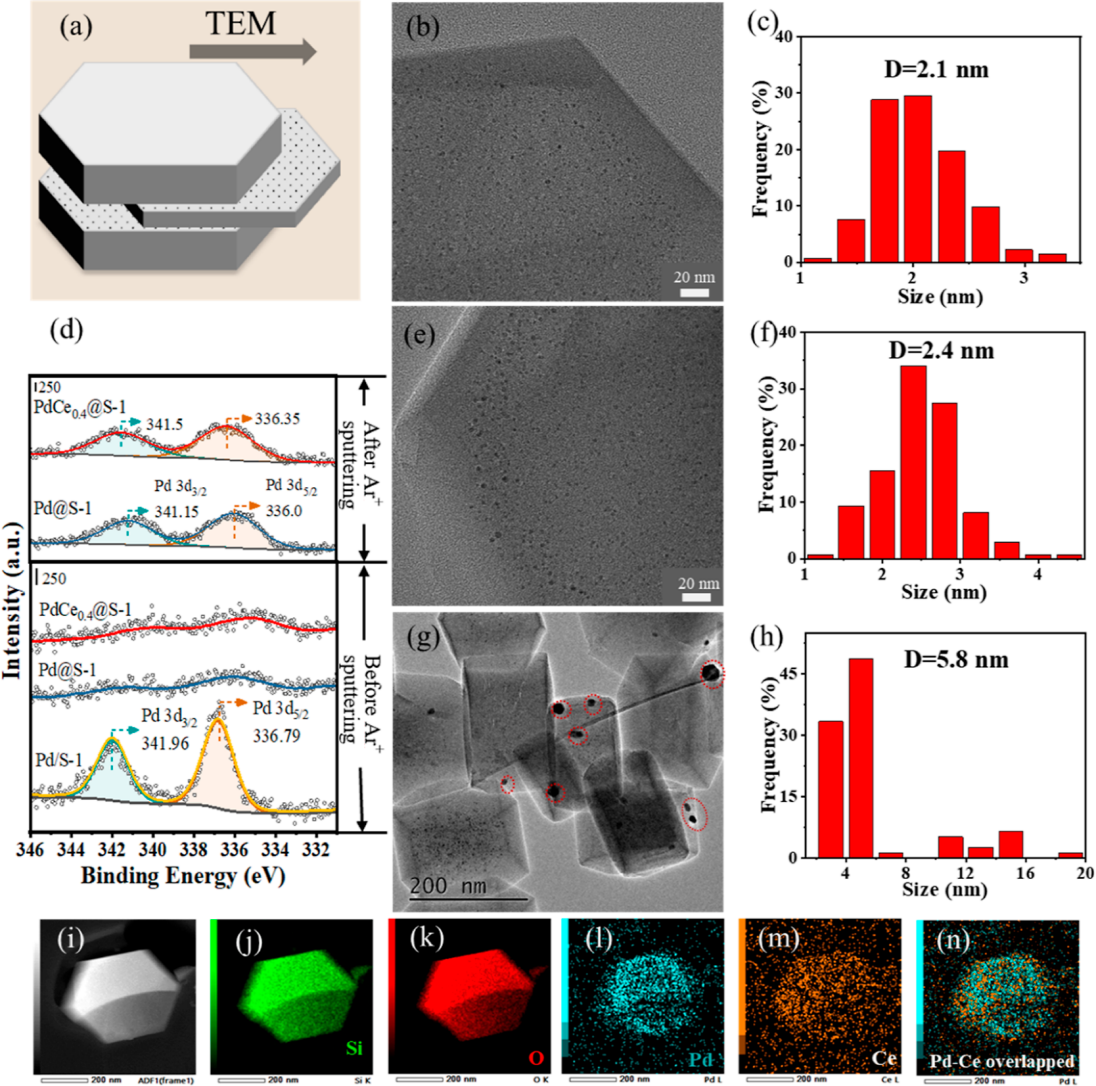
(a) Schematic illustration of the ultramicrotomy method. HRTEM images and size distributions of PdO or PdCe bimetallic oxide NPs for (b,c) Pd@S-1, (e,f) PdCe_0.4_@S-1, and (g,h) Pd/S-1. (d) Pd 3d XPS spectra of Pd/S-1, Pd@S-1, and PdCe_0.4_@S-1 before and after Ar^+^ ion sputtering. (i–n) HAADF-STEM images and elemental mappings for Si, O, Pd, and Ce of PdCe_0.4_@S-1 (ultramicrotomy).

XPS analysis was conducted to determine the chemical nature of Pd and Ce in these catalysts (Figures 2d and S4). While intense peaks at 336.79 and 341.96 eV attributed to Pd 3d_5/3_ and Pd 3d_3/2_ of PdO were observed in the Pd/S-1 sample prepared by the IWI method,^29^ no obvious Pd 3d signal was detected over Pd@S-1 and PdCe_0.4_@S-1 with similar Pd loadings as Pd/S-1. This finding further proves the complete confinement of PdO NPs and bimetallic Pd–Ce oxide NPs inside the S-1 zeolites for the two catalysts (Pd@S-1 and PdCe_0.4_@S-1). After argon ion sputtering, which was used to obtain the valence of Pd and Ce atoms within zeolite, clear peaks appeared at 336.0 (Pd 3d_5/2_) and 341.15 eV (Pd 3d_3/2_), suggesting the exposure of PdO species that were detectable by XPS (Figure 2d). Interestingly, compared to Pd@S-1, the Pd 3d_5/2_ and 3d_3/2_ positions of PdCe_0.4_@S-1 (336.35 and 341.5 eV) shifted to higher values, which can be ascribed to strong interactions between PdO and CeO_2_, leading to more electron deficiency of the Pd atoms.^20^ The predominant presence of PdO, widely accepted as the active phase for CH_4_ activation,^30,31^ is thus expected to favor the complete CH_4_ oxidation reaction.

The TEM images shown in Figure S5 confirm the typical hexagonal morphology of S-1 crystals, with a size of 200–400 nm observed in all the catalysts. To identify the exact locations of PdO NPs in S-1 (i.e., Pd@S-1 and PdCe_0.4_@S-1), an ultramicrotomy method (Figure 2a) was adopted to prepare specimen for TEM analysis. Specifically, the zeolite crystals embedded in resin were cut into 90 nm thick ultrathin slices by ultramicrotome, and thereby the exposed cross-sections of the zeolite crystal were analyzed to obtain information about the spatial distribution of metal species inside the crystals.^32^ The ultramicrotomy HRTEM images (Figure 2b,e) verifiably depict that the ultrasmall PdO NPs and Pd–Ce bimetallic oxide NPs were uniformly dispersed throughout the S-1 zeolites in Pd@S-1 and PdCe_0.4_@S-1, respectively. The corresponding average particle sizes of the metal oxides (PdO or Pd–Ce bimetallic oxide) were obtained by measuring more than 200 particles and found to be around 2.1 and 2.4 nm for Pd@S-1 and PdCe_0.4_@S-1, respectively (Figure 2c,f). By stark contrast, the image of PdO NPs in Pd/S-1 prepared by the IWI method was severely sintered and agglomerated with an uneven size distribution (Figure 2g,h), with some PdO particles even larger than 10 nm (red circles). The above HRTEM analysis signifies that the in situ encapsulation method could inhibit Pd aggregation and improve Pd dispersion. CO chemisorption experiments revealed a sequence of Pd dispersion (Table 1 and Figure S6) following: PdCe_0.4_@S-1 (26.2%) > Pd@S-1 (19.7%) > Pd/S-1 (15.2%), in good agreement with the TEM results. Elemental distribution in the PdCe_0.4_@S-1 (ultramicrotomy) was further measured using HAADF-STEM and EDX mapping (Figure 2i–n). Both Pd and Ce elements were evenly located at nearly identical positions, suggesting that both of Pd and Ce atoms were encapsulated into the zeolite matrix. Line scan analysis presents similar signal intensities of Pd and Ce (Figure S7), implying the formation of a bimetallic Pd–Ce oxide phase in the PdCe_0.4_@S-1 catalyst.

### 3.2 Catalytic Performance in Complete CH_4_ Oxidation

**Figure 3:**
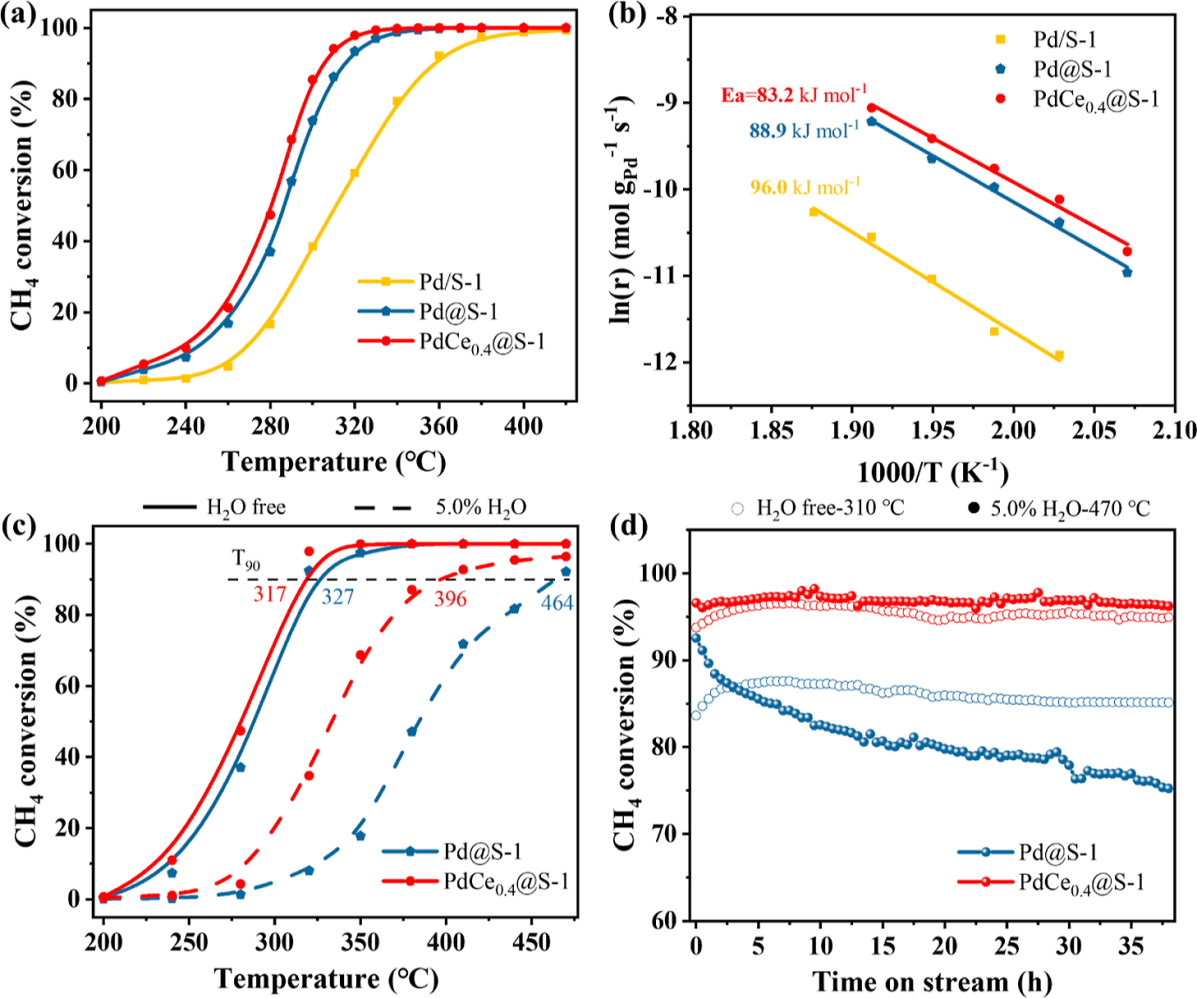
(a) CH_4_ conversion, (b) Arrhenius plots (reaction conditions: 1.0 vol % CH_4_, 20 vol % O_2_, balanced with N_2_, GHSV = 60,000 mL g_cat_^–1^ h^–1^), (c) water resistance test, and (d) long-term stability without H_2_O at 310 °C and with H_2_O at 470 °C over Pd@S-1 and PdCe_0.4_@S-1 (reaction conditions: 1.0 vol % CH_4_, 20 vol % O_2_, with or without 5.0% H_2_O, balanced with N_2_, GHSV = 60,000 mL g_cat_^–1^ h^–1^).

The catalytic activities of all the Pd catalysts for complete CH_4_ oxidation were measured and compared in Figures 3a and S8 and S9. Figure S10 shows the reaction temperatures required to achieve CH_4_ conversions of 10, 50, 90, and 100%, denoted as *T*_10_, *T*_50_, *T*_90_, and *T*_100_, respectively. The reaction rates, apparent activation energies (Ea), and TOF values are presented in Figure 3b and Table 2. As shown in Figure 3a, Pd@S-1, containing confined PdO NPs (with *T*_100_ at 370 °C), showed a much better activity than Pd/S-1 (*T*_100_ = 430 °C), which has PdO NPs on the zeolite surface. Notably, Pd@S-1 also has a lower Ea (88.9 kJ mol^–1^) than that of Pd/S-1(96.0 kJ mol^–1^), emphasizing the importance of the confinement effect in CH_4_ oxidation. According to TEM and CO chemisorption (Table 1), the in situ encapsulation method significantly increased Pd dispersion compared with the IWI method, leading to a higher density of accessible Pd sites in Pd@S-1, which are intrinsically beneficial for activating CH_4_ molecules.

**Table 2:** Catalytic Performance of the Obtained Pd Catalysts in Complete CH_4_ Oxidation

samples	*T*_100_ (°C)	rate^@240 °C^ (mmol g_Pd_^–1^s^–1^)	TOF^@240 °C^ (10^–2^s^–1^)	Ea (kJ mol^–1^)
Pd/S-1	440	1.6	1.1	96.0
Pd@S-1	370	6.0	3.2	88.9
PdCe_0.4_@S-1	350	9.2	3.7	83.2
PdCe_2.2_@S-1	410	3.2	1.4	90.8
PdCe_0.4_/S-1	440	0.92		103.3

Co-confining a small amount of Ce further decreased the *T*_100_ of Pd@S-1. However, when an excess amount of Ce was added, it was found to be detrimental to CH_4_ oxidation, as indicated by the unexpected increase of *T*_100_. Ultimately, the optimal Ce/Pd molar ratio was found to be 0.4 (PdCe_0.4_@S-1), with a *T*_100_ of 350 °C and an Ea value of 83.2 kJ mol^–1^ (about 20 °C and 5.7 kJ mol^–1^ lower than those of Pd@S-1, respectively; Table 2). It should be noted that Ce@S-1 displayed hardly any CH_4_ oxidation activity below 440 °C, suggesting that pure Ce species confined in S-1 are not active in CH_4_ oxidation (Figure S9). At 240 °C, the reaction rate of PdCe_0.4_@S-1 was found to be 1.5 times higher than that of Pd@S-1, and the TOF value of PdCe_0.4_@S-1 (0.037 s^–1^) was also the highest among all the Pd catalysts (Table 2). On the other hand, PdCe_0.4_/S-1 prepared by the IWI method, despite having the same Ce/Pd molar ratio as that of PdCe_0.4_@S-1, showed much lower activity in CH_4_ oxidation (*T*_100_ = 440 °C). Furthermore, PdCe_0.4_@S-1 also exhibited better catalytic activity than as-prepared Pd/Al_2_O_3_, Pd/SSZ-13, and Pd/SiO_2_ catalysts (all with Pd loadings of 2 wt %) in Figure S9. These results indicate that the confinement effect of S-1 zeolite induces a synergy between Pd and Ce and decreases the energy barrier of CH_4_ oxidation. In fact, PdCe_0.4_@S-1 is one of the best catalysts for complete CH_4_ oxidation as compared to state-of-the-art Pd catalysts reported in the literature (Table S3).

The Pd catalysts were also evaluated at high temperatures or in the presence of water vapor to explore their potential for real world applications where such harsh reaction conditions are commonly encountered (Figures S11 and S12).^17,33^ As shown in Figure S11, the temperature tolerance of PdCe_0.4_@S-1, Pd@S-1, and Pd/S-1 was evaluated, starting from 300 to 600 °C and then decreasing back to 300 °C. PdCe_0.4_@S-1 and Pd@S-1 exhibited initially higher activity than Pd/S-1 as presented before (Figure 3a), demonstrating their outstanding temperature tolerance with the aid of a confined structure. TEM measurements were performed again on the Pd catalysts after the temperature tolerance tests. While a severe Pd sintering was observed over Pd/S-1 (with an increase in PdO particle size from 5.8 to 9.2 nm and the appearance of PdO NPs even larger than 20 nm; Figure S13), only slight increases in particle size were noted over Pd@S-1 (from 2.1 to 2.4 nm) and PdCe_0.4_@S-1 (from 3.0 to 3.1 nm), confirming the effectiveness of confinement in preventing the agglomeration of Pd species. In addition, cycling experiments showed that PdCe_0.4_@S-1 demonstrated similar activity over five cycles of CH_4_ oxidation (Figure S12). TEM images showed that the metal particle size of the used catalysts increased slightly from 2.4 to 2.7 nm, suggesting the excellent stability of the PdCe_0.4_@S-1 catalyst (Figure S14).

In the presence of water vapor (in the feed gas or from methane combustion), which is believed to accelerate the sintering of Pd-based catalysts,^34^ the increase of *T*_90_ was less significant in the case of PdCe_0.4_@S-1 (by 90, or 306 to 396 °C) than Pd@S-1 (by 135 °C, or from 329 to 464 °C), indicating a stronger water resistance of PdCe_0.4_@S-1 (Figure 3c). Interestingly, in the 40 h stability tests (Figure 3d), PdCe_0.4_@S-1 maintained its activity under both dry (310 °C, no water vapor) and wet (470 °C, 5.0% water vapor) conditions, while Pd@S-1 gradually deactivated under wet conditions with a decrease of CH_4_ conversion from 92 to 74% (although it displayed stable performance under dry conditions as well). It is known that high concentrations of water vapor could poison Pd catalysts by forming an inactive Pd(OH)_2_ phase.^16,35,36^ Therefore, the confinement effect of hydrophobicity in S-1 zeolite is not the main reason for the higher water tolerance of PdCe_0.4_@S-1 compared to Pd@S-1. Instead, the Pd–CeO_2_ interaction is believed to inhibit the reaction between Pd and water to generate Pd(OH)_2_ in PdCe_0.4_@S-1.^27^ As shown in Figure S15, the Pd particle size of the used Pd@S-1 (after the water resistance experiment) increased by more than 1 nm (from 2.1 to 3.1 nm), while the used PdCe_0.4_@S-1 only increased from 2.4 to 2.8 nm. This finding unambiguously indicates that the synergistic effect of Pd and CeO_2_ in zeolite plays an important role in prohibiting the poison of water vapor on the active the Pd phase.

### 3.3 Mechanistic Investigations

#### 3.3.1 Role of Ce Co-confining

**Figure 4:**
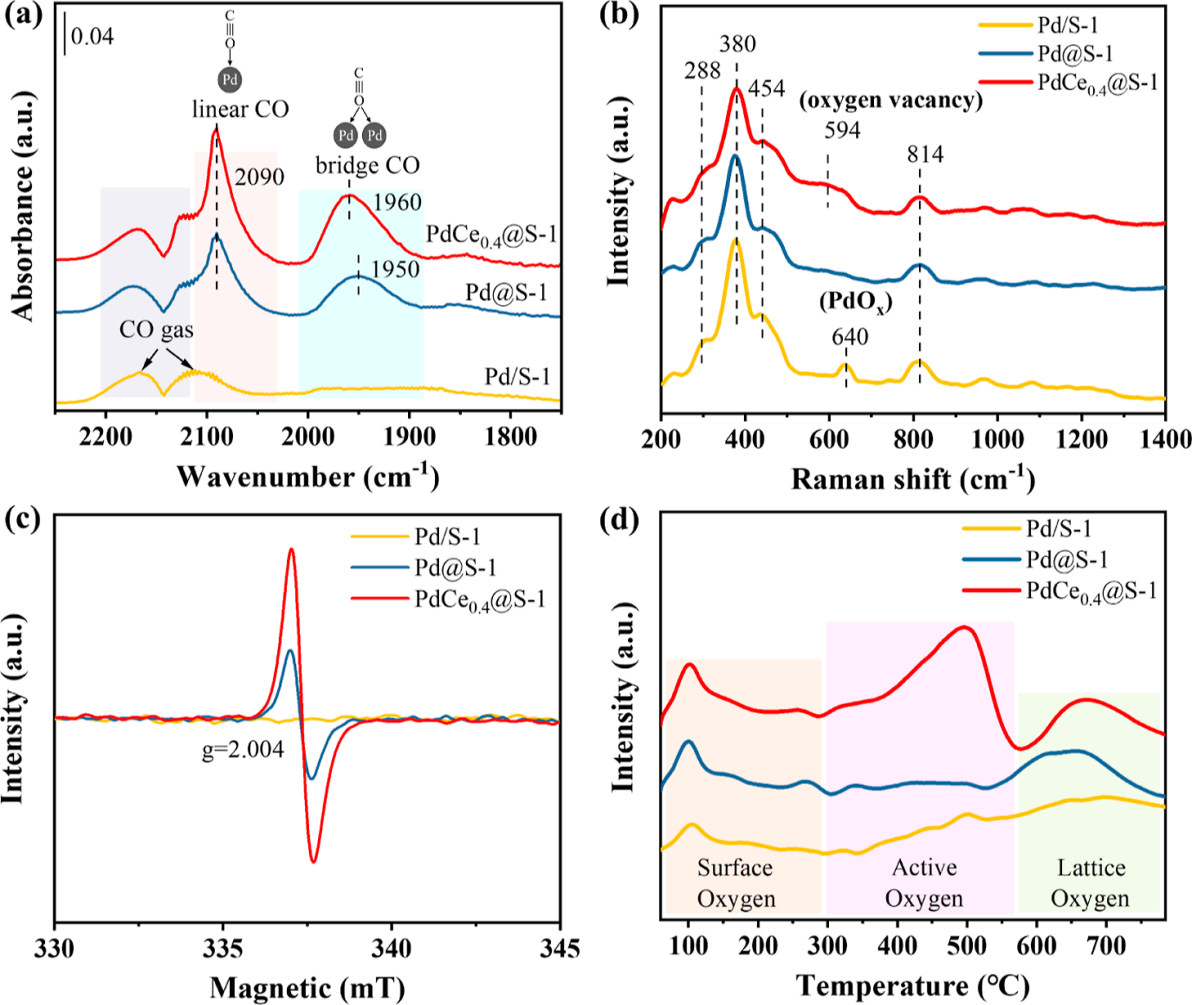
(a) CO-DRIFTS spectra (samples were pre-treated at 300 °C for 1 h in 10 vol % H_2_/Ar), (b) UV Raman, (c) EPR, and (d) O_2_-TPD of PdCe_0.4_@S-1 and related catalysts.

The abovementioned results of CH_4_ oxidation suggest a potential synergy between zeolite confinement and Ce co-confining in the PdCe_0.4_@S-1 catalyst. To gain more insight into the reaction mechanism, we performed in situ CO-DRIFTS, UV Raman, EPR, and O_2_-TPD analyses. H_2_ pre-treatment was performed before the in situ CO-DRIFTS test to reduce the Pd cations into a metallic state, which enhances the adsorption capacity of probe molecules (CO) and increases the signal intensity.^20^ This is because cationic Pd sites exhibit weaker interactions with CO molecules than metallic Pd sites.^37−40^ As shown in Figure 4a, only two bands located at 2112 and 2172 cm^–1^ for gaseous CO were observed over Pd/S-1, which is due to CO adsorption on large Pd particles without confinement. In contrast, clear CO adsorption bands were observed at 2090 and 1950 cm^–1^ over the encapsulated catalysts (i.e., Pd@S-1 and PdCe_0.4_@S-1) and can be assigned to the linear and bridging stretching vibrations of CO adsorption on Pd^0^ sites, respectively.^41^ The increased intensity of linear CO adsorption on PdCe_0.4_@S-1 is attributed to the formation of bimetallic Pd–Ce oxide NPs in the presence of a suitable amount of co-confined Ce species, which strengthened the CO adsorption on Pd sites.^42^ It is well documented that such bimetallic Pd–Ce oxide NPs could facilitate the cleavage of C–H of CH_4_ and enhance the activity of CH_4_ oxidation.^26^

**Figure 5:**
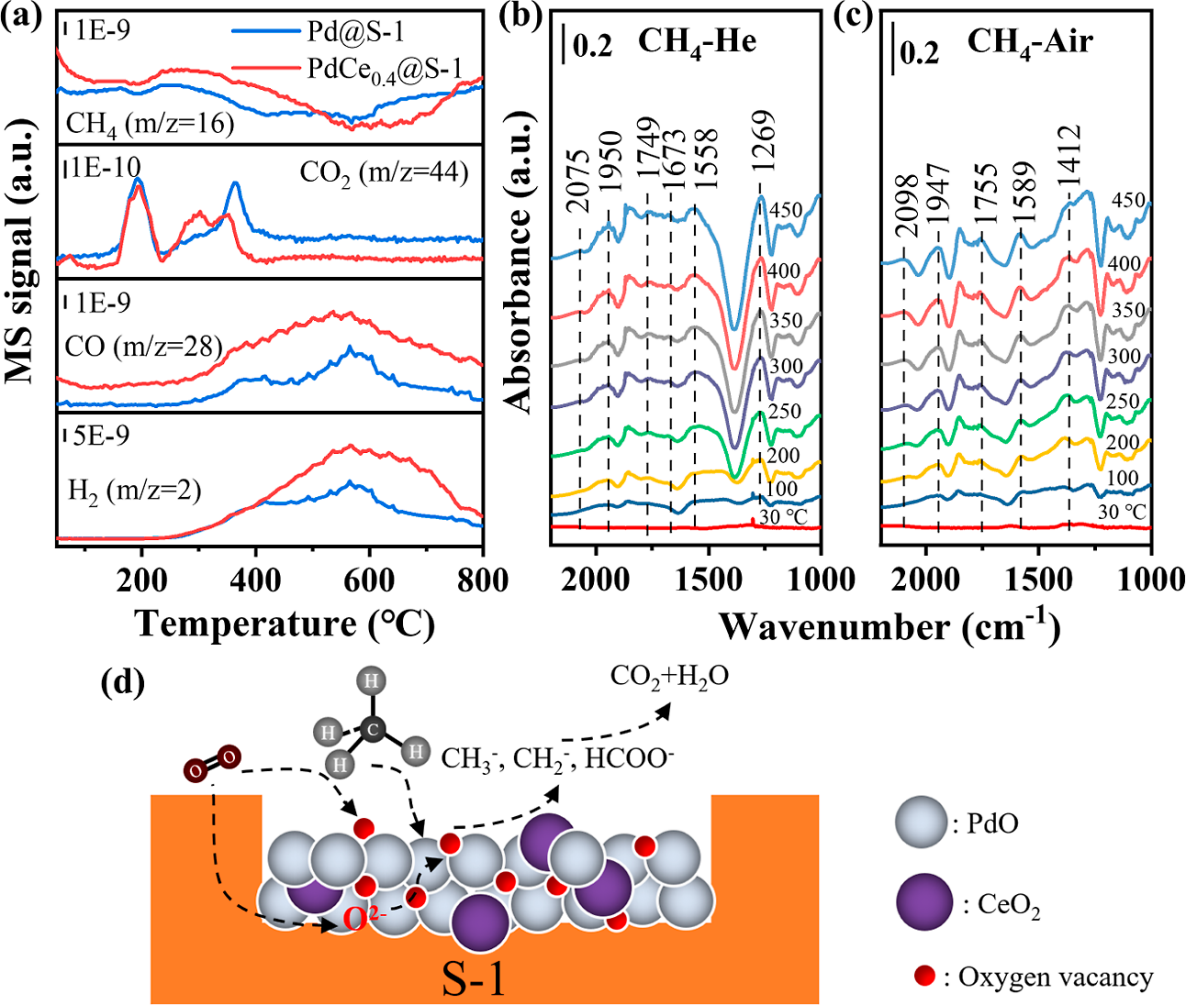
(a) CH_4_-TPR profiles of PdCe_0.4_@S-1 and Pd@S-1. In situ DRIFT spectra of PdCe_0.4_@S-1 under (b) 1 vol % CH_4_/He and (c) 1 vol % CH_4_/Air flows at various temperatures and (d) schematic illustration of the possible reaction pathway of CH_4_ oxidation over PdCe_0.4_@S-1.

In the UV Raman spectra (Figure 4b), peaks related to the S-1 zeolite framework (ca. 288, 380, 454, 814 cm^–1^) were detected in all the samples.^43^ For Pd/S-1, an additional band at ca. 640 cm^–1^ related to the B_1g_ vibration mode of the PdO phase was observed and can be attributed to the aggregation of the PdO phase in the catalyst,^44^ in line with TEM and CO DRIFTS observations. For PdCe_0.4_@S-1, a band at 594 cm^–1^ corresponding to a defect-induced (D) vibration mode was detected and is generally ascribed to oxygen vacancy sites in CeO_2_.^45,46^ EPR studies confirmed the presence of bulk oxygen vacancy defects in the PdCe_0.4_@S-1 catalysts. As shown in Figure 4c, a symmetrical signal at *g* = 2.004 ascribed to unpaired electrons in oxygen vacancies was visualized in the spectrum of PdCe_0.4_@S-1.^47,48^ The existence of oxygen vacancies is beneficial for the activation of gaseous O_2_ molecules to generate reactive oxygen species capable of cleaving C–H bonds in CH_4_.^49−51^ Accordingly, O_2_-TPD measurements were conducted to investigate the oxygen properties of PdCe_0.4_@S-1 and related catalysts. Indeed, in the O_2_-TPD profiles in Figure 4d, more intense oxygen desorption peaks were recorded at ca. 500 °C and ca. 650 °C, resulting from active species by the activation of surface lattice oxygen.^52^ These active oxygen species are generally considered to favor CH_4_ activation following the Mar–van Krevelen (M–vK) mechanism.^53^ In CH_4_-TPR experiments, we detected similar behaviors of CH_4_ consumption and CO_2_ formation in the absence of gas-phase O_2_ at temperatures below 250 °C over the PdCe_0.4_@S-1 and Pd@S-1 catalysts (Figure 5a). In the temperature range of 250–400 °C, CO formation was also detected, in addition to CH_4_ consumption and CO_2_ formation. The produced CO/CO_2_ likely resulted from the reaction between CH_4_ and active PdO species, as shown in the following equation
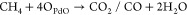
4

CH_4_ consumption at high temperatures (400–800 °C) was accompanied by the formation of CO and H_2_, which could be ascribed to the incomplete CH_4_ oxidation (CH_4_ + 2O → CO + 2H_2_O, CH_4_ + O → CO + 2H_2_) and/or the CH_4_ cracking (CH_4_ → C + 2H_2_).^54,55^ Notably, PdCe_0.4_@S-1 shows a lower CO_2_ formation temperature (centered at 320 °C) and a more intense peak of CO_2_ formation compared with Pd@S-1, which, according to O_2_-TPD, may result from a higher amount of active oxygen species after Ce co-confining.

#### 3.3.2 Reaction Pathway

In situ DRIFTS was combined with CH_4_-TPO to investigate the reaction intermediates during the catalytic CH_4_ oxidation over PdCe_0.4_@S-1, and the results are shown in Figures 5b,c and S16. Generally, the band intensity of CH_4_ (3016 cm^–1^) decreased with increasing temperature, while the bands at 1558 cm^–1^ ascribed to formate species at 1558 cm^–1^ appeared above 200 °C in CH_4_/He.^30^ Meanwhile, peaks attributed to the adsorbed bidentate carbonates (1673 cm^–1^) and bicarbonates (1269 cm^–1^) intermediates were also detected.^30,56^ Interestingly, broad bands centered at 1950 and 2075 cm^–1^ related to chemisorbed CO in bridging and linear forms, respectively, appeared and gradually increased in intensity with temperature, which is due to the direct reaction between CH_4_ and the O species in PdO. The likely reaction process over PdCe_0.4_@S-1 is: CH_4_ → CH_4_* → formate → bidentate carbonates/bicarbonates → CO → CO_2_. In a CH_4_ + air atmosphere, the bridge-adsorbed CO (1947 cm^–1^) and linear-adsorbed CO (2098 cm^–1^) bands also appeared (Figure 5c). Notably, the bands for adsorbed formate (1558 cm^–1^) and bidentate carbonates (1673 cm^–1^) intermediates vanished after the addition of air, while the bands for carboxylates (1589 cm^–1^) and carbonate (1412 cm^–1^) appeared,^30,57^ indicating an accelerated transformation of formate to carbonate by O_2_ injection. The reaction process thus changed to CH_4_ → CH_4_* → carbonate → carboxylates → CO → CO_2_.

Based on in situ DRIFTS, CH_4_-TPR, and O_2_-TPD results as well as published literature,^58,59^ we propose the reaction pathway of CH_4_ oxidation over PdCe_0.4_@S-1 following the M-vK mechanism (Figure 5d), where the activation of the first C–H bond is the rate-determining step.^50^ Initially, CH_4_ is adsorbed on the active sites of bimetallic Pd–Ce mixed oxide inside the S-1 crystal and activated to methyl species and surface hydroxyl groups. Subsequently, the CH_3_^–^ and CH_2_^–^ species are oxidized by the active oxygen species to form carbonate and carboxylate intermediates, which are further oxidized to chemisorbed CO. Finally, the chemisorbed CO is transformed to CO_2_ and H_2_O as the final products. Moreover, the diffusion of lattice oxygen from bulk or the adsorption of gas-phase O_2_ would refill the oxygen vacancies of PdCe_0.4_@S-1. It is known that the active lattice oxygen species play a crucial role when CH_4_ oxidation follows the M-vK mechanism.^52,53^ Therefore, PdCe_0.4_@S-1 with more active oxygen species exhibits superiority in the oxidation of CH_4_ to CO_2_ and H_2_O.

## 4 Conclusions

In summary, a bimetallic Pd–Ce oxide catalyst confined inside S-1 zeolite was successfully synthesized using a facile in situ encapsulation strategy and demonstrated superior catalytic activity (with a *T*_100_ of 350 °C) and thermal stability in complete CH_4_ oxidation. The confinement effect promoted the accessibility of highly dispersed active Pd sites and prevented sintering at high temperatures and/or in the presence of water vapor, while the introduction of Ce generated more oxygen vacancies and active oxygen species that are effective in CH_4_ activation and oxidation. The strong interaction between Pd and Ce within the hydrophobic S-1 zeolite protected the active PdO phase from transformation into inactive Pd(OH)_2_ in high-concentration water vapor, thus ensuring exceptional stability in CH_4_ oxidation. These findings provide a perspective for the rational design and development of active and stable catalysts for complete CH_4_ oxidation under harsh conditions.

## Supporting Information Available

Experimental details, additional catalyst characterization, additional catalytic performance, TEM images and size distribution of catalysts after reaction, additional in situ DRIFT spectra, additional information of catalysts, and comparison of different catalysts for methane combustion (PDF)
