# Supplementary material for: Unlocking High-Efficiency Methane Oxidation with Bimetallic Pd–Ce Catalysts under Zeolite Confinement
Source: ACS Environ Au. 2023 May 16;3(4):223–32. doi: 10.1021/acsenvironau.3c00008 (PMC10360205; doi:10.1021/acsenvironau.3c00008)
Supplement: Supplementary file 1 — vg3c00008_si_001.pdf [file vg3c00008_si_001.pdf]

Supporting Information

Unlocking High-Efficiency Methane Oxidation with Bimetallic Pd-Ce Catalysts under Zeolite Confinement

Xiaomai Chen[a], Xuefeng Shi[a], Peirong Chen[a], Bowen Liu[c], Meiyin Liu[a], Longwen Chen[b], Daiqi Ye[a], Xin Tu[c, *], Wei Fan[d, *], Junliang Wu[a, *]

[a] National Engineering Laboratory for VOCs Pollution Control Technology and Equipment, Guangdong Provincial Key Laboratory of Atmospheric Environment and Pollution Control, School of Environment and Energy, South China University of Technology, Guangzhou, 510006, China

[b] College of Light Chemical Industry and Materials Engineering, Shunde Polytechnic, Foshan, 528333, China

[c] Department of Electrical Engineering and Electronics, University of Liverpool, Liverpool L69 3GJ, UK

[d] Department of Chemical Engineering, University of Massachusetts-Amherst, Amherst, MA 01003, USA

*Corresponding authors: xin.tu@liverpool.ac.uk (Xin Tu); wfan@ecs.umass.edu (Wei Fan); ppjl@scut.edu.cn (Junliang Wu)

Table of Contents

1. Experimental details

2. Additional catalyst characterization

3. Additional catalytic performance

4. TEM images and size distribution of catalysts after reaction

5. Additional *in situ* DRIFT spectra

6. Additional information of catalysts

7. Comparison of different catalysts for methane combustion

8. References

1. Experimental details

The metal loading was determined using inductively coupled plasma - mass spectrometry (ICP-MS) on a Horiba Ultra 2 instrument equipped with a photomultiplier tube detector. Powder X-ray diffraction (PXRD) patterns were obtained using a Bruker D8 Advance X diffractometer equipped with a Cu Kα source at ambient conditions. The data were collected at a scan rate of 10 ° min^{-1} over a scanning range of scattering angle 2θ from 10° to 80°. Nitrogen physisorption isotherms were measured on an ASAP 2020 automatic surface analyzer (Micromeritics, USA). Prior to the measurements, all samples were degassed at 250 °C under vacuum for 10 h. The specific surface area was calculated from the Brunauer–Emmett–Teller (BET) model, and the t-plots model was used to determine the external surface area and micropore volume. Thermal gravimetric analysis was conducted on thermogravimetric analyzer STA 449 system (Netzsc, Germany). The sample was heated from room temperature to 800 °C in air at a heating rate of 10 °C min^{-1}.

Transmission electron microscopy (TEM) images were obtained using a JEOL JEM-2100HR. The sample was dispersed in ethanol by ultrasound, and a few drops of the suspension were tiled on a copper grid. High resolution Transmission electron microscopy (HRTEM) images were obtained using a FEI Tecnai G2 f20 microscope operating at an accelerating voltage of 200 kV. In addition, to confirm the exact location of Pd and Ce within the S-1 zeolite, the resin-embedded catalysts were ultra-microtomed and sliced into 80-nm-thick sections using a diamond knife, following the procedures described in previous studies.[1,2] To further prove the spatial distribution of Pd and Ce in the PdCe0.4@S-1 catalyst, high-angle annular dark-field scanning TEM (HAADF-STEM), corresponding energy-dispersive X-ray (EDX) mapping images (EX-37001), and line scans were performed.

X-ray photoelectron spectroscopy (XPS) analysis was conducted on a Physical Electronics Quantum 2000 equipped with monochromatic Al Kα source (Kα = 1486.6 eV) and a charge neutralizer. The binding energies of all the elements were calibrated using the C 1s line at 284.8 eV. Moreover, to determine the valence of Pd and Ce atoms within the zeolite, depth-profile analysis was performed using an XPS system with an argon ion etch gun. The test was conducted using 3000 eV of ion energy and 180 s of etch time.

The Raman test was performed using a LabRAM Aramis Raman spectrometer (HYJ, France). The excitation light source has a wavelength of 325 nm (ultraviolet) and the scanning range is 200-1400 cm^{-1}. Low-temperature electron paramagnetic resonance (EPR) was

measured using a JEOL electron spin resonance spectrometer.

The O_2 temperature-programmed desorption (O_2-TPD) experiment was carried out using a Micromeritics Autochem II 2920. First, the catalysts were pretreated in a He flow (50 mL min^{-1}) at 300 °C for 1 h. After cooling to 60 °C, the flow was switched to 5.0 vol % O_2/He and kept for 1 h to adsorb O_2. Finally, the temperature was increased to 800 °C in He (50 mL min^{-1}) at a heating rate of 10 °C min^{-1}, while recording the thermal conductivity detector (TCD) signal. CO chemisorption was performed using the Micromeritics AutoChem II 2920. Prior to adsorption, a 50 mg sample was pretreated at 300 °C for 1 h in 10 vol % H_2/Ar and cooled down to 30 °C in Ar. Pulse chemisorption was carried out with pulses of 0.5243 mL of 10 vol % CO/He until saturation, which corresponds to 2.14×10^{-6} mol of saturated CO amount (CO_S). The Pd dispersion was estimated using the following equation:

Pd dispersion (%) = (mole of exposed Pd atom) / (mole of total Pd atom of catalyst) = (mole of adsorption of CO) / (mole of total Pd atom of catalyst).

The adsorption of CO was estimated using the following equation:
adsorption of CO (mol) = (peak area of CO adsorption) / (peak area of saturated CO) × CO_S (peak area of CO was obtained by integrating the data in Figure S5).

The CH_4 temperature-programmed reduction (CH_4-TPR) experiment was carried out using the same apparatus that was equipped with a HIDEN HPR-20 mass spectrometer (MS). First, 50 mg of the catalyst was pretreated at 300 °C for 40 min in Ar (50 mL min^{-1}), then cooled to room temperature. The TPR test was conducted by increasing the temperature of the samples to 800 °C at a heating rate of 10 °C min^{-1} in 1.0 vol % CH_4/Ar (30 mL min^{-1}). The signal of CH_4 (m/z=16), as well as CO_2, CO and H_2 (m/z=44, 28, 2) were recorded online by the MS.

In situ diffuse reflectance infrared Fourier transform (DRIFT) spectra were obtained using a Nicolet Nexus spectrometer equipped with a liquid nitrogen-cooled mercury cadmium telluride (MCT) detector. The sample cell featured KBr windows. DRIFT spectra of the catalysts were collected with a resolution of 4 cm min^{-1} and 64 scans in absorbance units. For the DRIFT spectra of CO adsorption, the samples were pretreated with 10 vol % H_2/Ar (30 mL min^{-1}) at 300 °C for 1 h. Then, 1.0 vol % CO/Ar (30 mL min^{-1}) was passed through the cell at 25 °C, and the IR spectra were monitored. Similarly, DRIFT spectra of CH_4 adsorption on the catalysts were measured. For the CH_4 temperature-programmed oxidation with O_2 and without

O_2 experiments, prior to each experiment, the catalyst was heating to 550 °C for 1 h under a He flow to remove surface impurities and then cooled to 30 °C to obtain a background spectrum. Afterward, 1.0 vol % CH_4/He (30 mL min^{-1}) was flowed to the IR cell, and the temperature was increased from 30 °C to 450 °C at various intervals while allowing for adsorption for 30 min. The IR spectra were monitored in real-time.

2. Additional catalyst characterization

TGA curves

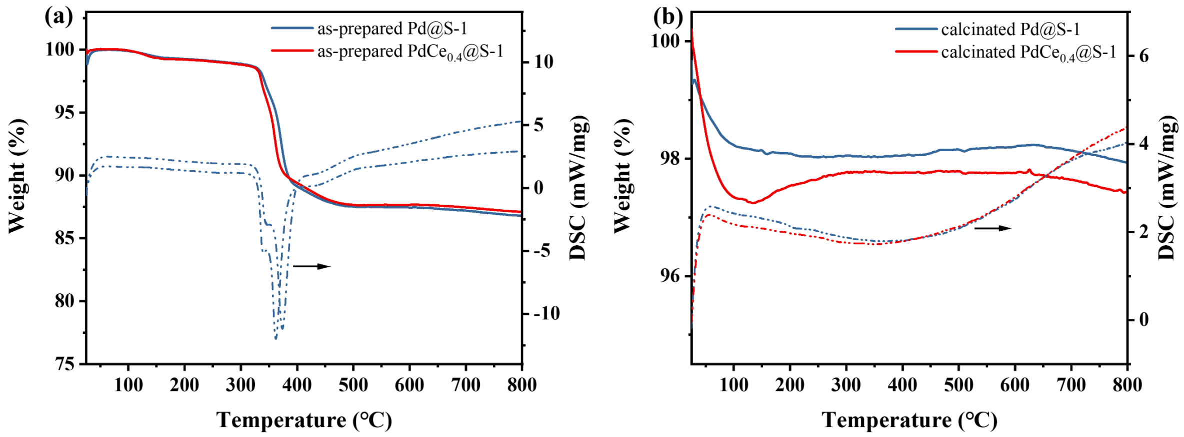

Figure S1. TGA curves of (a) as-prepared (b) calcined Pd@S-1 and $PdCe_{0.4}$@S-1.

Notes: The thermogravimetry differential thermal analysis (TG-DTA) curves of uncalcined representative Pd@S-1 and PdCe0.4@S-1 catalysts showed an endothermic peak at around 350 °C (Figure S1a), which can be attributed to the oxidation of organic ligands and templates in air.[3] Moreover, the TG-DTA curves of the calcined Pd@S-1 and $PdCe_{0.4}$@S-1 catalysts showed a weight loss of only about 2% due to surface adsorbed water below 100 °C, and this weight loss hardly decreased after 100 °C (Figure S1b). This indicates that air calcination can effectively remove organic ligands and templates.

PXRD patterns

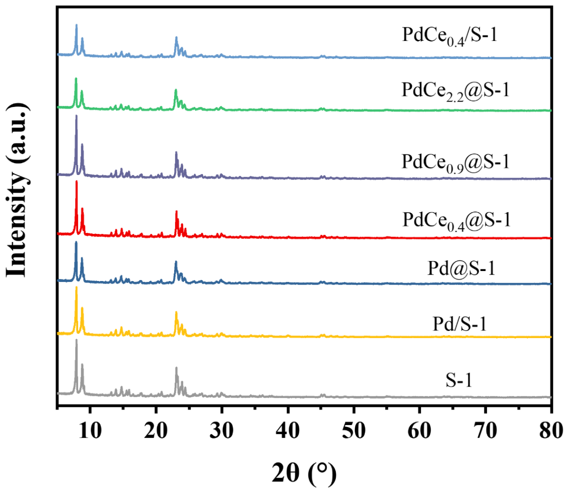

Figure S2. PXRD patterns of $PdCe_{0.4}$@S-1 and related catalysts.

N_2 adsorption/desorption isotherms

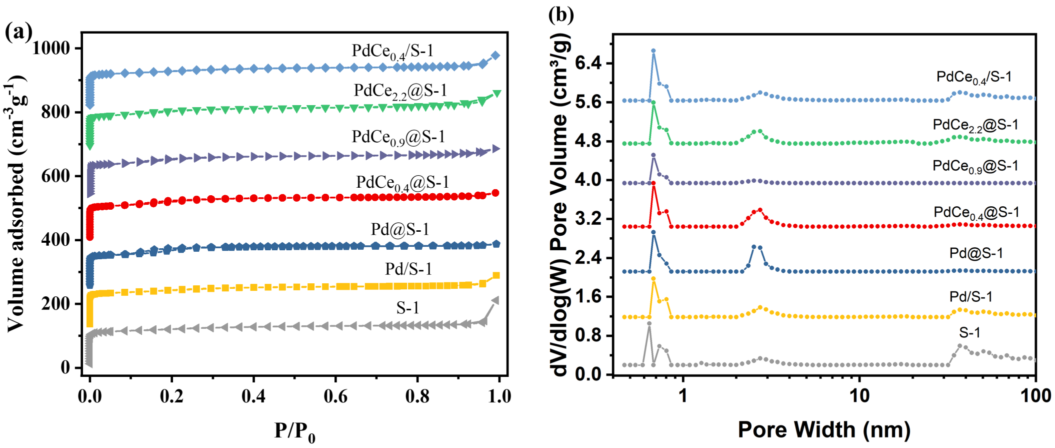

Figure S3. (a) N_2 adsorption/desorption isotherms and (b) pore size distribution of $PdCe_{0.4}$@S-1 and related catalysts.

Note: According to the results of BET surface area (Figure S3a and Table S2), a slight increase in surface area was noticed in the Pd contained samples (389 m^2 g^{-1} of Pd@S-1 and 387 m^2 g^{-1} of Pd/S-1) compared to pure S-1 (373 m^2 g^{-1}), indicating that the introduction of Pd NPs nearly did not affect the porosity of S-1. However, after the introduction of Ce, the specific surface area slightly decreased with the increase in Ce content. The sample with the highest level of Ce ($PdCe_{2.2}$@S-1) had a surface area of 370 m^2 g^{-1}, smaller than that of Pd@S-1. This decrease may be due to the presence of CeO_2 on the outer surface of the catalyst, formed by the excessive addition of Ce, as confirmed by the reduced S_{exter} (182 m^2 g^{-1} of $PdCe_{2.2}$@S-1, 261 m^2 g^{-1} of Pd@S-1). Additionally, the micropore volume of Pd@S-1 and $PdCe_{0.4}$@S-1 decreased by around 49.2% and 30.0%, respectively, compared to pure S-1 support (from 0.13 to 0.066 and 0.091 cm^3 g^{-1}), implying that the volume of micropores was partially occupied. This result indicates that the metal NPs were confined in the channels of the nanosized S-1 zeolite, but there were still enough channels for the diffusion of reactants and products.[2,4]

Additional XPS data

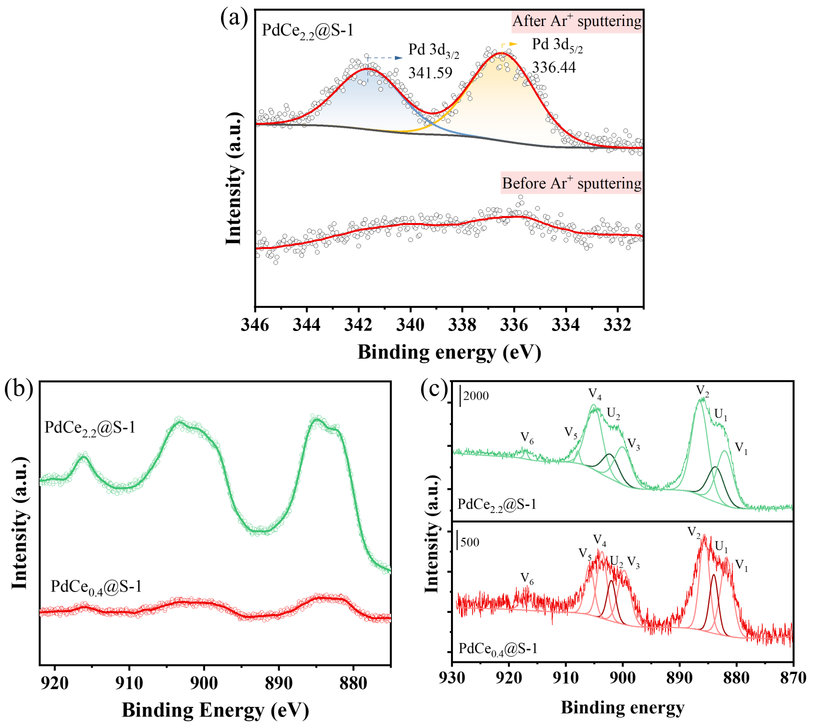

Figure S4. (a) Pd 3d of $PdCe_{2.2}$@S-1 before and after of Ar^+ sputtering, Ce 3d XPS spectra of $PdCe_{0.4}$@S-1 and $PdCe_{2.2}$@S-1 (b) before and (c) after Ar^+ sputtering.

Note: As shown in Figure S4b, a stronger intensity of Ce 3d is observed over $PdCe_{2.2}$@S-1 compared to $PdCe_{0.4}$@S-1. This suggests that although the Pd species were encapsulated within S-1 zeolites of $PdCe_{2.2}$@S-1 catalyst, partial CeO_2 aggregated on the outer surface of S-1 due to the excessive addition of Ce. This result is consistent with the slightly decreasing specific surface area. After Ar^+ sputtering, Figure S4c shows six peaks of V_6 (917.1 eV), V_5 (905.9 eV), V_4 (903． 7 eV), V_3 (899.8． eV), V_2 (885.7 eV) and V_1 (881.8 eV) ascribed to Ce^{4+} species, and two peaks of U_2(902.0 eV) and U_1(884.0 eV) attributed to Ce^{3+} species over $PdCe_{0.4}$@S-1, as documented in the literature.[5]

TEM images

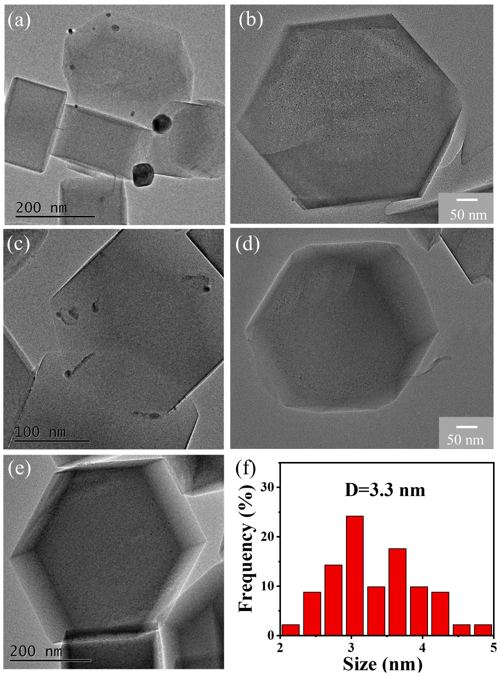

Figure S5. TEM images of (a) Pd/S-1, (b) Pd@S-1, (c) $PdCe_{0.4}$/S-1 (d) $PdCe_{0.4}$@S-1 and (e) $PdCe_{2.2}$@S-1 catalysts. (f) Size distribution of $PdCe_{2.2}$@S-1.

CO chemisorption

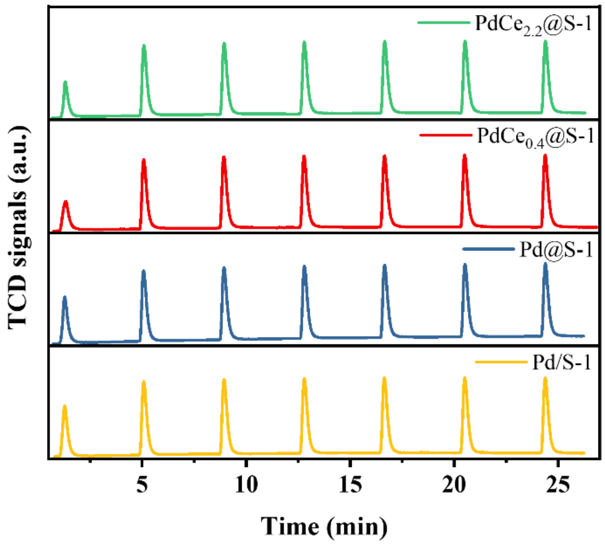

Figure S6. CO chemisorption of various catalysts.

Note: It is generally well-documented that higher metal dispersion signifies smaller metal particle size[6, 7]. However, in comparison to Pd@S-1, catalysts containing Ce with the same

confined structure exhibit a larger amount of exposed Pd atoms, but with a larger particle size. Therefore, it is reasonable to conclude that the particle size of $PdCe_x$@S-1 catalysts gradually increases with increasing Ce loading due to the formation of Pd-CeO_2 mixed oxide particles within $PdCe_x$@S-1, rather than purely Pd NPs. Additionally, the higher Pd dispersion of $PdCe_x$@S-1 catalysts indicates that the form of Pd-CeO_2 interaction further enhances the number of accessible Pd sites. However, as a result of the higher Ce loading, $PdCe_{2.2}$@S-1 displays a larger particle size (3.3 nm) (Figures S4e and S4f), but less Pd dispersion compared to $PdCe_{0.4}$@S-1, which is likely due to the over-accumulation of excessive CeO_2 surrounding the Pd NPs.

HAADF-STEM images and line scan

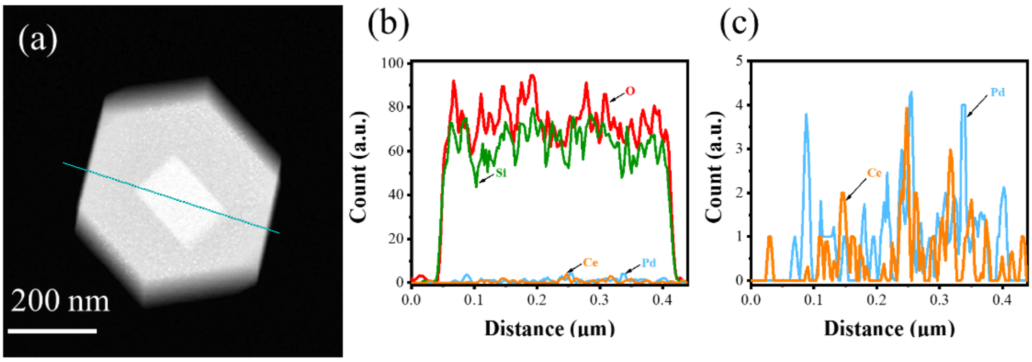

Figure S7. (a) HAADF-STEM images, (b-c) line scan of $PdCe_{0.4}$@S-1.

3. Additional catalytic performance

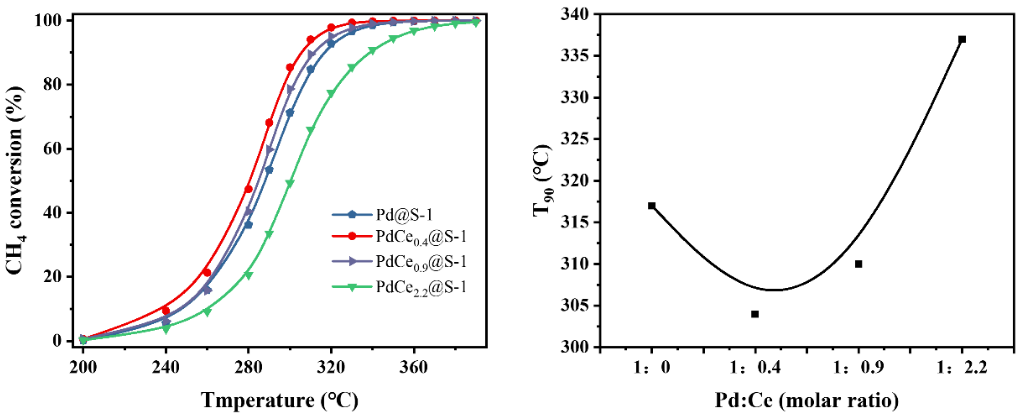

Figure S8. (a) Methane conversion and (b) corresponding T_{90} profiles over $PdCe_x$@S-1 with various Pd/Ce ratios. Reaction conditions: 1 vol % CH_4, 20 vol % O_2, and balance with N_2, gas hourly space velocity (GHSV) = 60,000 mL g_{cat}^{-1} h^{-1}.

Note: However, the excessive addition of Ce resulted in reduced activity of $PdCe_{2.2}$@S-1 compared to Pd@S-1. XPS and BET analyses revealed that the excess CeO_2 mainly exists on the outer surface of the zeolite and does not form Pd-CeO_2 bimetallic NPs. This excess CeO_2 may occupy some of the pores, partially hindering contact between the reactants and the active Pd sites. That could be the reason for the decreased activity of $PdCe_{2.2}$@S-1 with excessive Ce addition.

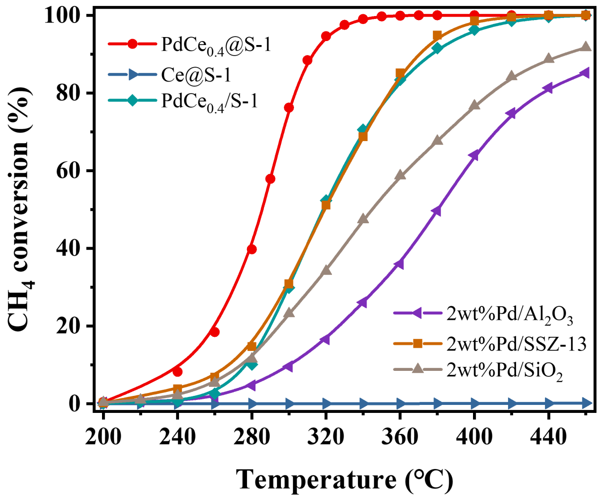

Figure S9. Methane conversion over $PdCe_{0.4}$/S-1, Ce@S-1 and commercial Pd/Al_2O_3. (Al_2O_3, SSZ-13 and SiO_2 are commercial support, and 2 wt %Pd/Al_2O_3, 2 wt % Pd/SSZ-13 and 2 wt % Pd/SiO_2 were prepared using the incipient wetness impregnation method) Reaction conditions: 1 vol % CH_4, 20 vol % O_2, balanced with N_2, GHSV = 60,000 mL g_{cat}^{-1} h^{-1}.

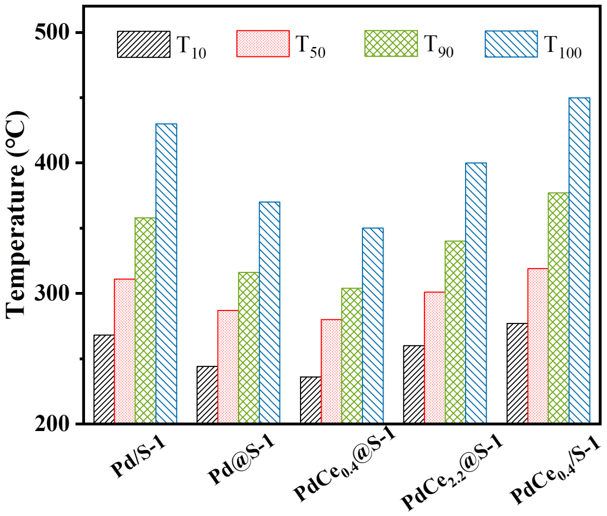

Figure S10. The detailed activity of T_{10}, T_{50}, T_{90} and T_{100} over $PdCe_{0.4}$@S-1 and related catalysts.

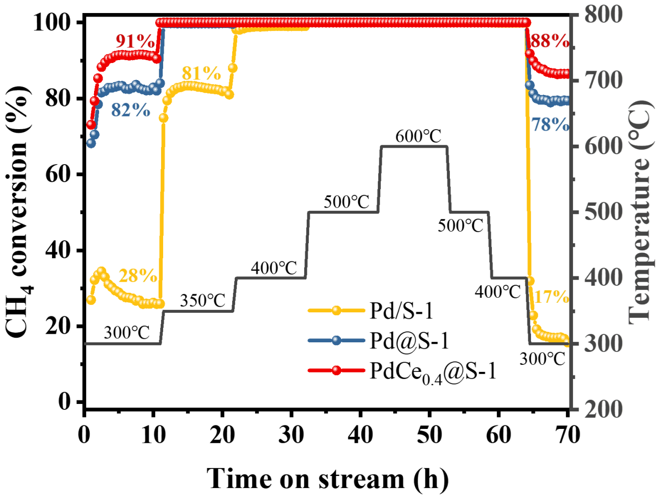

Figure S11. Temperature tolerance over Pd/S-1, Pd@S-1 and PdCe$_{0.4}$@S-1 (Reaction conditions: 1.0 vol % CH_4, 20 vol % O_2, balanced with N_2, GHSV = 60,000 mL g_{cat}^{-1} h^{-1}).

Note: Figure S11 shows the CH_4 conversions at various temperatures within the temperature tolerance range of PdCe$_{0.4}$@S-1, Pd@S-1 and Pd/S-1, starting from 300 °C and increasing up to 600 °C and then decreasing back to 300 °C. As shown earlier in Figure 3a, PdCe$_{0.4}$@S-1 and Pd@S-1 exhibit initially higher activity compared to Pd/S-1. Notably, after cooling down to 300 °C, the methane conversion over PdCe$_{0.4}$@S-1 and Pd@S-1 only slightly decreased from 91% to 88% and 82% to 78%, respectively. In contrast, the conversion over Pd/S-1 decreased from 28% to 17%, indicating the exceptional temperature tolerance of PdCe$_{0.4}$@S-1 and Pd@S-1 with the help of their confined structures.

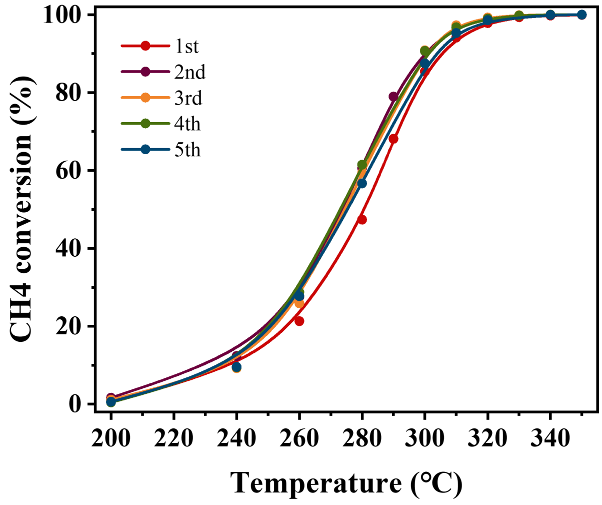

Figure S12. Durability test. Reaction conditions: 1 vol % CH_4, 20 vol % O_2, balanced with N_2, GHSV = 60,000 mL g_{cat}^{-1} h^{-1}.

4. TEM images and size distribution of catalysts after reaction

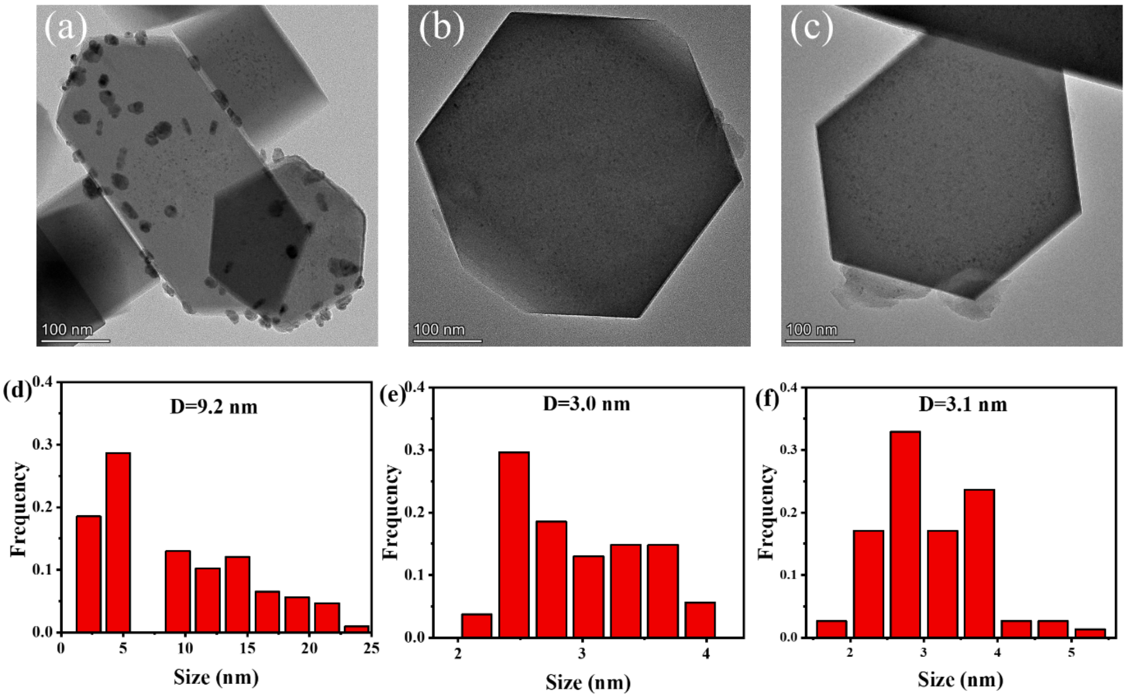

Figure S13. TEM images and size distributions of Pd/S-1 (a, d), Pd@S-1 (b, e), $PdCe_{0.4}$@S-1 (c, f) after treatment at 700 °C under reaction conditions for 8 h.

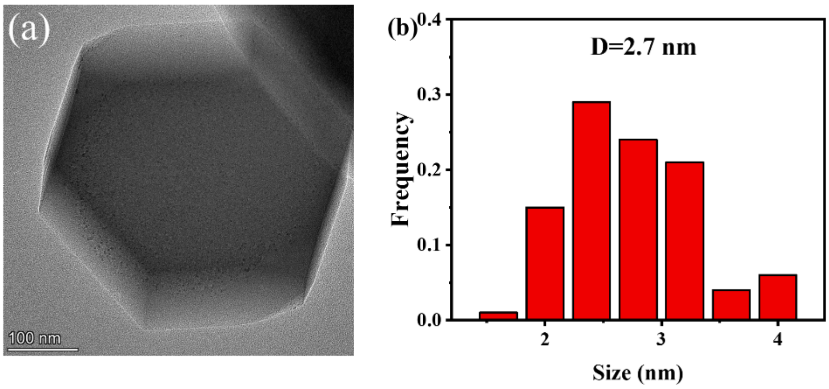

Figure S14. TEM image (a) and size distribution (b) of $PdCe_{0.4}$@S-1 after durability test.

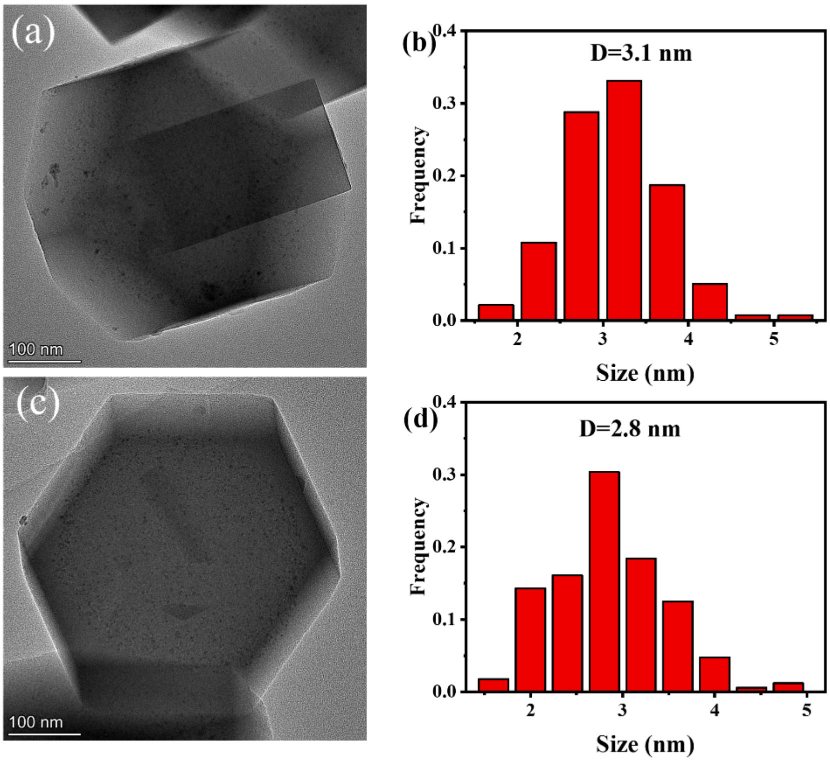

Figure S15. TEM images and size distributions of Pd@S-1 (a, b) and PdCe$_{0.4}$@S-1 (c, d) after water resistance experiment.

5. Additional *in situ* DRIFT spectra

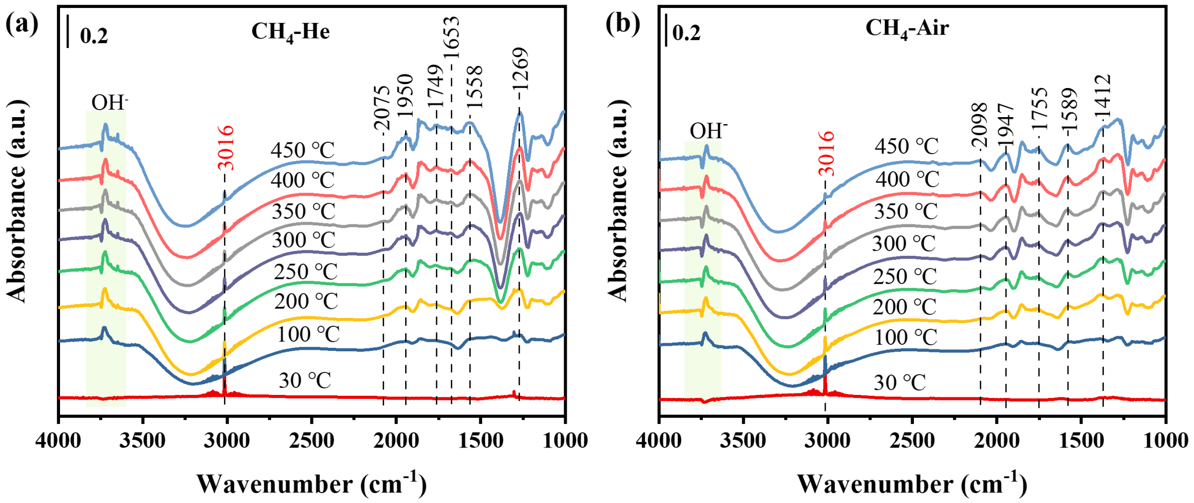

Figure S16. *In situ* DRIFT spectra of PdCe$_{0.4}$@S-1 under (a) 1 vol % CH_4/He and (b) 1 vol % CH_4/Air flows at various temperatures.

Note: The absorption bands at 3016 and 1300 cm^{-1} correspond to the asymmetric stretching

vibration ν_{as} (C–H) and deformation vibration δ(C–H) of CH_4, respectively.[8] Additionally, the signals around 1750 and 3500-3800 cm^{-1} are attributed to the hydroxyl groups (OH^-) adsorbed on the surface, which originate from CH_4 oxidation.

6. Additional information of catalysts

Pd and Ce content of catalysts

Table S1. Pd and Ce contents of the catalysts.

Samples	Pd contents (wt %)[a]	Ce contents (wt %)[a]	Ce/Pd ratios
Pd@S-1	1.00	-	-
$PdCe_{0.4}$@S-1	1.09	0.58	0.4
$PdCe_{0.9}$@S-1	1.15	1.40	0.9
$PdCe_{2.2}$@S-1	1.07	3.20	2.2
$PdCe_{0.4}$/S-1	0.88	0.40	0.4
Ce@S-1	-	0.63	-
Pd/S-1	1.07	-	-

a: Measured by ICP-MS

Structural properties

Table S2. The structural properties of $PdCe_{0.4}$@S-1 and related catalysts.

Samples	S_{BET} (m^2 g^{-1})[a]	S_{micro} (m^2 g^{-1})[a]	S_{exter} (m^2 g^{-1})[a]	V_{total} (cm^3 g^{-1})[b]	V_{micro} (cm^3 g^{-1})[a]
Pd@S-1	389	127	261	0.20	0.066
$PdCe_{0.4}$@S-1	386	175	211	0.22	0.091
$PdCe_{0.9}$@S-1	371	165	205	0.21	0.086
$PdCe_{2.2}$@S-1	370	188	182	0.22	0.097
$PdCe_{0.4}$/S-1	372	224	148	0.21	0.12
Pd/S-1	387	205	182	0.22	0.11
S-1	373	250	123	0.21	0.13

[a] t-plot method

[b] Single point desorption total pore volume of pores, $P/P_0 = 0.975$

7. Comparison of different catalysts for methane combustion

Table S3. Comparison of different catalysts for methane combustion.

Catalysts	Pd (wt %)	Gas composition (vol %)			GHSV (mL g^{-1} h^{-1})	T_{100} (°C)	Ref
		CH_4	O_2	H_2O			
0.6Pd@S-1	0.6	1	16	-	50,000	380	2
0.6Pd@S-1	0.6	1	16	4.5	50,000	405	2
$Pd_{0.8}Ni_{0.2}$@S-1	0.8	1	20	-	24,000	T_{90} = 303	9
Pd/Na-MOR	1.0	1	4	-	70,000 (h^{-1})	400	10
Pd/Na-MOR	1.0	1	4	5	70,000 (h^{-1})	450	10
PdCo@S-1	0.6	1	20	-	30,000	400	4
PdCo@S-1	0.6	1	20	5	30,000	435	4
Pd@CeO_2/H-Al_2O_3	1.0	0.5	2	-	200,000	400	11
Pd-CeNW@SiO_2	1.5	1	21	-	36,000	350	12
Pd-CeNW@SiO_2	1.5	1	21	5	36,000	450	12
PdO/CeO_2@ HZSM-5	0.93	0.5	10	3	30,000	550	13
Pd@IM-S-1	1.8	1	21	-	36,000	T_{90} = 318	14
$PdCe_{0.4}$@S-1	1.0	1	21	-	60,000	350	This work
$PdCe_{0.4}$@S-1	1.0	1	21	5	60,000	T_{90} = 396	This work

8. References

(1) Zecevic, J.; Vanbutsele, G.; de Jong, K. P.; Martens, J. A. Nanoscale intimacy in bifunctional catalysts for selective conversion of hydrocarbons. *Nature* **2015**, *528*, 245-248.

(2) Wang, W.; Zhou, W.; Li, W.; Xiong, X.; Wang, Y.; Cheng, K.; Kang, J.; Zhang, Q.; Wang, Y. In-situ confinement of ultrasmall palladium nanoparticles in silicalite-1 for methane combustion with excellent activity and hydrothermal stability. *Applied Catalysis B: Environmental* **2020**, *276*.

(3) Wang, N.; Sun, Q.; Bai, R.; Li, X.; Guo, G.; Yu, J. In situ confinement of ultrasmall Pd clusters within nanosized silicalite-1 zeolite for highly efficient catalysis of hydrogen generation. *J. Am. Chem. Soc.* **2016**, *138*, 7484-7487.

(4) Tang, X.; Lou, Y.; Zhao, R.; Tang, B.; Guo, W.; Guo, Y.; Zhan, W.; Jia, Y.; Wang, L.; Dai, S.; et al. Confinement of subnanometric PdCo bimetallic oxide clusters in zeolites for methane complete oxidation. *Chem. Eng. J.* **2021**, *418*, 129398.

(5) Paparazzo, E. XPS studies of damage induced by X-ray irradiation on CeO_2 surfaces. *Surface science* **1990**, *234*, L253-L258.

(6) Chen, J.; Zhong, J.; Wu, Y.; Hu, W.; Qu, P.; Xiao, X.; Zhang, G.; Liu, X.; Jiao, Y.; Zhong, L.; et al. Particle Size Effects in Stoichiometric Methane Combustion: Structure–Activity Relationship of Pd Catalyst Supported on Gamma-Alumina. *ACS Catal.* **2020**, *10*, 10339-10349.

(7) Kon, K.; Hakim Siddiki, S. M. A.; Shimizu, K.-i. Size- and support-dependent Pt nanocluster catalysis for oxidant-free dehydrogenation of alcohols. *Journal of Catalysis* **2013**, *304*, 63-71.

(8) Scarano, D.; Bertarione, S.; Spoto, G.; Zecchina, A.; Areán, C. O. FTIR spectroscopy of hydrogen, carbon monoxide, and methane adsorbed and co-adsorbed on zinc oxide. *Thin Solid Films* **2001**, *400*, 50-55.

(9) Zhang, Z.; Sun, L.; Hu, X.; Zhang, Y.; Tian, H.; Yang, X. Anti-sintering Pd@silicalite-1 for methane combustion: Effects of the moisture and SO_2. *Appl. Surf. Sci.* **2019**, *494*, 1044-1054.

(10) Losch, P.; Huang, W.; Vozniuk, O.; Goodman, E. D.; Schmidt, W.; Cargnello, M. Modular Pd/Zeolite Composites Demonstrating the Key Role of Support Hydrophobic/Hydrophilic Character in Methane Catalytic Combustion. *ACS Catalysis* **2019**, *9*, 4742-4753.

(11) M. Cargnello, J. J. D. J., J. C. Hernández Garrido, K. Bakhmutsky, T. Montini,; J. J. Calvino Gámez, R. J. G., P. Fornasiero. Exceptional Activity for Methane Combustion over Modular Pd@CeO_2 Subunits on Functionalized Al_2O_3. *Science* **2012**, *337*.

(12) Peng, H.; Rao, C.; Zhang, N.; Wang, X.; Liu, W.; Mao, W.; Han, L.; Zhang, P.; Dai, S.

Confined Ultrathin Pd-Ce Nanowires with Outstanding Moisture and SO_2 Tolerance in Methane Combustion. *Angewandte Chemie International Edition* **2018**, *57*, 8953-8957.
(13) Dai, Q.; Bai, S.; Lou, Y.; Wang, X.; Guo, Y.; Lu, G. Sandwich-like PdO/CeO_2 nanosheet@HZSM-5 membrane hybrid composite for methane combustion: self-redispersion, sintering-resistance and oxygen, water-tolerance. *Nanoscale* **2016**, *8*, 9621-9628.
(14) Peng, H.; Dong, T.; Yang, S.; Chen, H.; Yang, Z.; Liu, W.; He, C.; Wu, P.; Tian, J.; Peng, Y.; et al. Intra-crystalline mesoporous zeolite encapsulation-derived thermally robust metal nanocatalyst in deep oxidation of light alkanes. *Nature Communications* **2022**, *13*, 295.
